# Association of Zinc Deficiency with Iron Deficiency Anemia and its Symptoms: Results from a Case-control Study

**DOI:** 10.7759/cureus.3811

**Published:** 2019-01-02

**Authors:** Jehan S Abdo Soliman, Ahmed Y Amer, Jehan S Abdo Soliman

**Affiliations:** 1 Internal Medicine, Zagazig University Hospitals, Zagazig, EGY

**Keywords:** iron deficiency anemia, zinc deficiency, case control

## Abstract

Iron deficiency anemia (IDA) is the most common type of anemia. Impaired iron absorption can be caused by a decrease in trace elements as zinc, which is found in the structure of enzymes that coordinate or catalyst iron metabolism. The aim of this study is to detect if zinc deficiency is associated with IDA and to determine the effect of associated zinc deficiency on symptoms of IDA in adult IDA patients. This case-control study included 30 IDA patients with matched healthy control group (n = 30) for age and sex. For each subject, the parameters were evaluated: hemoglobin (Hb); mean corpuscular volume (MCV), total iron binding capacity (TIBC), serum iron, serum ferritin (SF). Serum zinc levels were measured by atomic absorption method with the PerkinElmer Analyst. Symptoms attributed to iron deficiency or depletion, defined as fatigue, cardiopulmonary symptoms, mental manifestations, epithelial manifestations, and neuromuscular symptoms were recorded. Serum zinc levels were lower in IDA patients (43.4 ± 7.9 mg/dL) than in the control subjects (94.7 ± 16.75 mg/dL; p < 0.0001). Zinc deficiency was associated with worse cardiovascular symptoms (p = 0.04), epithelial symptoms (p = 0.027), and restless leg syndrome (p < 0.001) in patients with IDA. Measurement of zinc levels should be considered for IDA patients. With the help of our study, iron and zinc supplementation instead of only iron replacement may be considered in cases of iron deficiency particularly, in patients with severe epithelial dysfunctions. Further studies are still needed to evaluate the benefit of zinc and iron supplementation in IDA patients.

## Introduction

Iron deficiency is the most common nutritional deficiency worldwide affecting about 1.48 billion people [1]. Women and young children are most commonly affected in developing countries. Moreover, it is the only nutrient deficiency which is also significantly prevalent in industrialized countries [2, 3]. Iron deficiency anemia (IDA) is associated with weakness, shortness of breath, and serious health risks including abnormal mental and motor development. Although rare, glossitis or dysphagia may be identified at presentation [4, 5]. Treatment of IDA is a major public health goal, especially in developing countries.

Iron deficiency can co-exist with deficiencies of other trace elements such as zinc, which is more frequently encountered in developing countries. Zinc acts as the catalyst in iron metabolism in the activity of alpha-aminolevulinic acid dehydratase enzyme, which plays a role in heme synthesis [6]. Zinc is found in the structure of the growth factor independent 1B (Gfi-1B) zinc finger protein, which functions as a regulator in erythroid cell growth by modulating gene expression specific to erythroid series [7, 8].

However, there are no enough studies about the serum zinc levels in patients with IDA. So, the aim of this study is to evaluate serum zinc levels in patients with IDA and also to determine the effect of associated zinc deficiency on symptoms of iron deficiency anemia.

## Materials and methods

We conducted a case-control study including randomly selected 30 cases between 18 and 50 years of age with IDA who had presented to our outpatient clinics at the hematology department, Zagazig University Hospitals between October 2016 and March 2017. We included 30 healthy persons with the same age range served as the control group. Patients with history of (1) macrocytic anemia, (2) chronic liver disease, (3) chronic renal disease, (4) infection or blood transfusion within one month were excluded.

Peripheral blood smear examinations were conducted in the Hematology Department. Those with predominantly microcytic indices (mean corpuscular volume (MCV) < 80 fL) and microcytic hypochromic picture on peripheral smear and hypochromic indices (Mean corpuscular hemoglobin (MCH) < 26 pg/cell) were considered as probably having iron deficiency anemia, which was then confirmed by low serum ferritin levels below 20 µg/dL and low serum-free iron [9]. Serum ferritin levels were measured using the chemiluminescence technique on Advia Centaur XP, Germany. Zinc deficiency was defined as levels below 70 ug/dL [10-11]. Serum zinc levels were measured by atomic absorption method with the PerkinElmer Analyst 800, Germany.

Statistical analyses were performed using the statistical software program, SPSS, for Windows version 20.0 (SPSS; Chicago, IL, USA). Results were given as mean ± standard deviation (±SD) and parametric independent t test was performed to detect statistical differences between the two groups when the variables showed normal distribution by Kolmogorov-Smirnov test. Pearson’s correlations were used for correlation analyses. A value of p < 0.05 was considered statistically significant. We calculated the sensitivity, specificity, positive and negative predictive values for zinc level as a predictor for IDA.

## Results

We included 30 healthy individuals and 30 patients with iron deficiency in this study. The demographic characteristics and biochemical findings of the patients are shown in Table 1.

**Table 1:** Demographic characteristics and biochemical findings of patients and healthy control groups. IDA: Iron deficiency anemia; MCV: Mean corpuscular volume; TIBC: Total iron binding capacity.

	Patients with IDA (n = 30)	Healthy controls (n = 30)
Age (years)	31 ± 7.67	32.3 ± 5.9
Gender (Female)	13 (43.3%)	12 (40%)
Hemoglobin (g/dL)	9.4 ± 0.44	13.4 ± 0.09
MCV (fL)	75.6 ± 2.3	82.3 ± 2.8
Ferritin (μg/l)	35.1 ± 19.1	126 ± 24.4
Iron (μg/dL)	44.1 ± 19.1	91.7 ± 8.3
TIBC (μg/dL)	287.9 ± 53.1	253.2 ± 43.5
Zinc (mg/dL)	43.4 ± 7.9	94.7 ± 16.75

**Figure 1:**
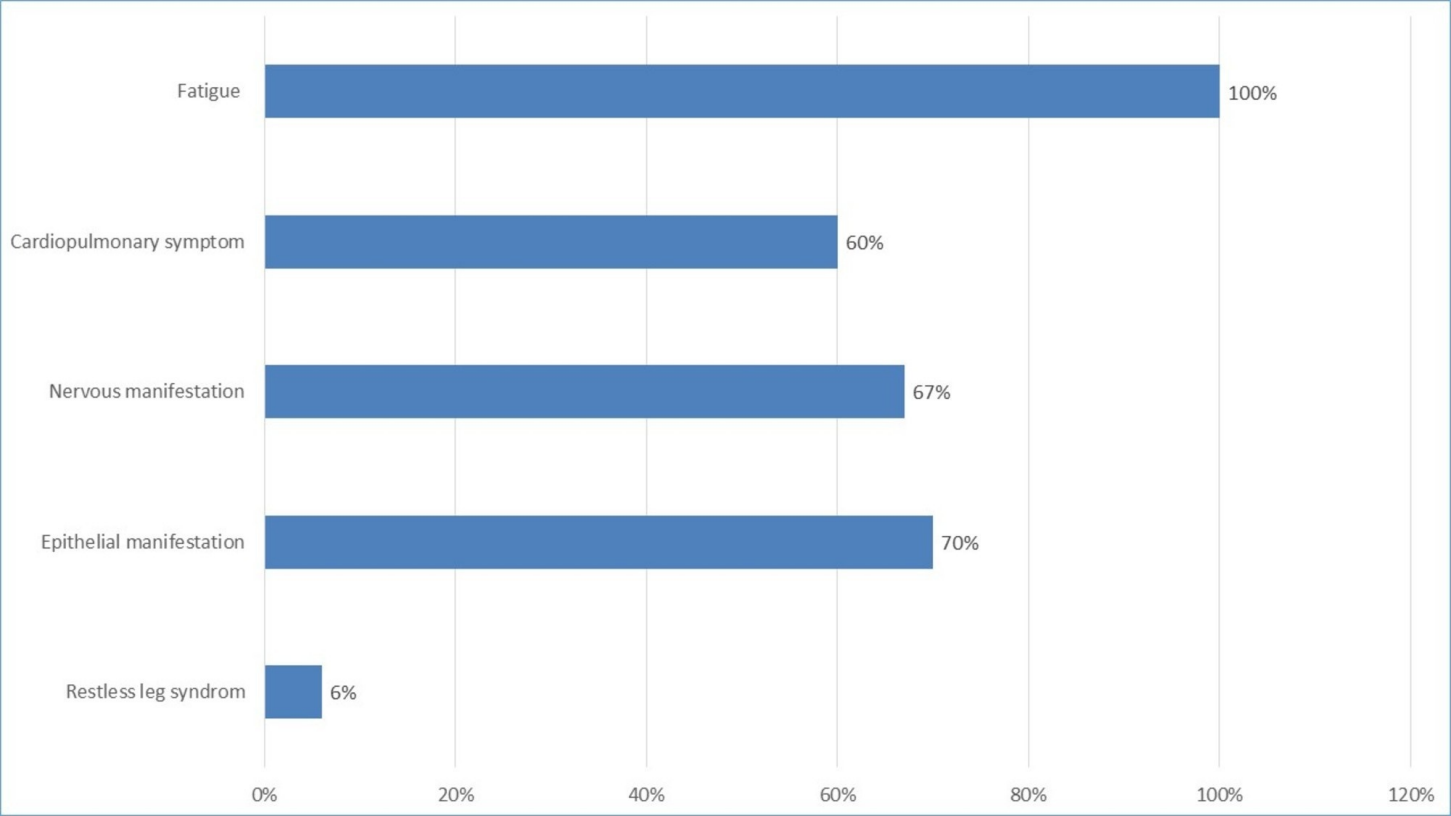
Symptoms distribution among iron deficiency anemia (IDA) patients.

There was no significant difference between the groups in terms of age or gender (p > 0.05). There was a statistically significant difference between the groups in terms of Hb (p < 0.001), MCV (p < 0.001), serum iron (p = 0.001), total iron binding capacity (p = 0.008), and ferritin (p < 0.001). Serum zinc levels were lower in IDA patients (43.4 ± 7.9 mg/dL) than in the control subjects (94.7 ± 16.75 mg/dL). The difference was highly statistically significant between the two groups (p < 0.0001). Figure 1 shows the frequency distribution of the most common presenting symptoms in IDA patients.

The most commonly reported symptom associated with IDA were fatigue (100%) followed by cardiopulmonary symptom (tachypnea, tachycardia, palpitation, 60%), epithelial manifestation (dermatitis, glossitis and diarrhea, 70%), mental manifestation (irritability, headache and amnesia, 67%), and restless leg syndrome (RLS, 6%). There has been a significantly lower zinc level in patients with iron deficiency who had cardiovascular symptoms as tachycardia (p = 0.04), epithelial symptoms as dermatitis (p = 0.027), and restless leg syndrome (p = 0.000) (Table 2).

**Table 2:** Symptom of IDA in relation to zinc deficiency. IDA: Iron deficiency anemia; RLS: Restless leg syndrome.

Symptom of IDA	Mean zinc level (±SD)	Statistics	P value
Tachycardia (n = 8)	39.3 (5.4)	-2.15	0.041*
No tachycardia (n = 22)	45.1 (8.6)		
Palpitation (n = 3)	37 (2.4)	-2.64	0.011*
No palpitation (n = 27)	45.3 (8.3)		
Dermatitis (n = 9)	40.3 (7.14)	2.301	0.027*
No dermatitis (n = 21)	46.638 (5.1)		
RLS (n = 1)	43.2	-15.58	0.001*
No RLSs (n = 29)	47.8 (8.03)		
Fatigue (n = 30)	43.5 (2.1)	-	-
No fatigue (n = 0)	-		

Our results revealed a significant correlation between the serum zinc level and MCV, MCH, total iron binding capacity (TIBC), serum iron, and serum ferritin level in patients with IDA, but not in healthy control (Table 3).

**Table 3:** Correlation analysis between serum zinc level and other parameters in IDA patients and healthy controls. IDA: Iron deficiency anemia; MCV: Mean corpuscular volume; MCH: Mean corpuscular hemoglobin; TIBC: Total iron binding capacity.

	Patients with IDA (n = 30)		Healthy controls (n = 30)	
	Correlation	P value	Correlation	P value
Age (years)	-0.36	0.04	-0.11	0.53
Hemoglobin	0.15	0.41	0.21	0.72
MCV	0.08	0.65	0.28	0.12
MCH	0.13	0.50	0.43	0.01
Iron level	-0.17	0.34	0.98	0.0001
Ferritin level	-0.27	0.15	0.96	0.0001
TIBC	-0.11	0.54	0.99	0.0001

## Discussion

Zinc and iron are the most important trace elements in homeostasis. Iron and zinc have important roles in heme structure, iron absorption, iron transport and exhibit competitive inhibition in transport and bio-availability [12-13]. Zinc acts as a catalyst in heme metabolism being part of GFi-1B zinc finger protein structure, which is a major regulator in erythroid cell growth by modulating gene expression specific to erythroid series, performs transcriptional regulation during erythropoiesis [3, 4]. The association between zinc deficiency and iron deficiency may be due to nutritional insufficiency of both elements or malabsorption.

Iron deficiency is the most common nutritional deficiency worldwide affecting women and young children in developing countries. As there are multiple etiological factors like malnutrition, low socioeconomic status, high-fiber diet, milk allergies, and parasitic infections, it is not a wonder to find a significant association between zinc deficiency and iron deficiency [5]. Several studies in the literature have been conducted to elaborate the fact that zinc deficiency and iron deficiency are correlated. In 1997, Ece et al. measured the serum zinc levels in children with iron deficiency. The serum zinc levels were lower in the IDA group than the control group (p = 0.017) [6]. Likewise, Gürgöze et al. [7] reported that the serum zinc concentration in the IDA group was found to be significantly lower than those in the control group (p < 0.05). Most of the published studies were conducted on children while, in 2016, Özhan et al. conducted a study including women with IDA (aged 18 to 60 years). They found that the serum zinc level was lower in the IDA group, which was statistically significant [8].

Moreover, Kelkitli et al. showed that there was a statistically significant difference between the two groups in serum zinc levels, which were lower in the IDA patients (103.51 μ/dL) compared to the healthy control groups (256.92 μ/dL) [9].

Our results were in accordance with the previous reports and we showed that the serum zinc levels were lower in adult patients with IDA (43.4 mg/dL) compared with the control subjects (94.7 mg/dL). The difference was statistically significant between the two groups (p < 0.0001).

The present study showed that iron deficiency anemia can be associated with tachycardia, tachypnea, headache, palpitation, dermatitis, glossitis, RLS, irritability, and fatigue. The most commonly reported symptom related to IDA was fatigue (100%). Similar result was revealed by Kelkitli et al. [9]. They reported that all the patients with IDA had complaints of fatigue (100%). The second most common reported symptoms were the cardio-pulmonary symptoms (60%) including tachypnea (26.7%), tachycardia (23.3%), and palpitation (10%). The epithelial symptoms of IDA were aggravated by the presence of zinc deficiency. Our results showed a frequency of dermatitis and glossitis by 30% and 40% respectively in IDA patients. It was proved that zinc deficiency may cause gastrointestinal and skin epithelial lesions [10-13]. Our results were consistent with Kelkitli et al. as they found that the epithelial manifestations were seen in 53.5% of patients with IDA and 88% of patients with IDA and zinc deficiency [9]. Also, they reported that the prevalence of RLS was 24%, which is approximately six times higher than that seen in our study (3%) and is higher than that seen in the general population by nine folds [14]. The serum zinc level was lower in symptomatic patients than non-symptomatic ones and the difference was statistically significant.

A significant correlation between the serum zinc level and MCV, MCH, TIBC, serum iron, and serum ferritin level has been identified in patients with IDA. These data support the hypothesis that coexistence of zinc and iron deficiencies can exaggerate the degree and the symptoms of IDA.

## Conclusions

We suggest that serum zinc levels should be evaluated in adults with IDA. With the help of our studies, iron and zinc treatment instead of only iron replacement may be considered in cases of iron deficiency particularly in patients with severe epithelial dysfunctions. Further studies are still needed to evaluate the benefit of zinc and iron supplementation in IDA patients with aggravated symptoms.

## References

[1] Espinoza A, Le Blanc S, Olivares M, Pizarro F, Ruz M, Arredondo M (2012). Iron, copper, and zinc transport: inhibition of divalent metal transporter 1 (DMT1) and human copper transporter 1 (hCTR1) by shRNA. Biol Trace Elem Res.

[2] Arredondo M, Martínez R, Núñez MT, Ruz M, Olivares M (2006). Inhibition of iron and copper uptake by iron, copper and zinc. Biol Res.

[3] Osawa M, Yamaguchi T, Nakamura Y (2002). Erythroid expansion mediated by the Gfi-1B zinc finger protein: role in normal hematopoiesis. Blood.

[4] Angelova MG, Petkova-Marinova TV, Pogorielov MV, Loboda AN, Nedkova-Kolarova VN, Bozhinova AN (2014). Trace element status (iron, zinc, copper, chromium, cobalt, and nickel) in iron-deficiency anaemia of children under 3 years. Anemia.

[5] Önal S, Nazıroğlu M, Çolak M, Bulut V, Flores-Arce MF (2011). Effects of different medical treatments on serum copper, selenium and zinc levels in patients with rheumatoid arthritis. Biol Trace Elem Res.

[6] Ece A, Uyanik BS, Işcan A, Ertan P, Yiğitoğlu MR (1997). Increased serum copper and decreased serum zinc levels in children with iron deficiency anemia. Biol Trace Elem Res.

[7] Gürgöze MK, Olçücü A, Aygün AD, Taskin E, Kiliç M (2006). Serum and hair levels of zinc, selenium, iron, and copper in children with iron-deficiency anemia. Biol Trace Elem Res.

[8] Özhan O, Erdem N, Aydoğdu İ, Erkurt A, Kuku İ (2016). Serum zinc levels in iron deficient women: a case-control study. Turk J Hematol.

[9] Kelkitli E, Ozturk N, Aslan NA (2016). Serum zinc levels in patients with iron deficiency anemia and its association with symptoms of iron deficiency anemia. Ann Hematol.

[10] Skrovanek S, DiGuilio K, Bailey R (2014). Zinc and gastrointestinal disease. World J Gastrointest Pathophysiol.

[11] Scrimgeour AG, Condlin ML (2009). Zinc and micronutrient combinations to combat gastrointestinal inflammation. Curr Opin Clin Nutr Metab Care.

[12] Saper RB, Rash R (2009). Zinc: an essential micronutrient. Am Fam Physician.

[13] Oken E, Duggan C (2002). Update on micronutrients: iron and zinc. Curr Opin Pediatr.

[14] Allen RP, Auerbach S, Bahrain H, Auerbach M, Earley CJ (2013). The prevalence and impact of restless legs syndrome on patients with iron deficiency anemia. Am J Hematol.

