# Correction: Association of Zinc Deficiency with Iron Deficiency Anemia and its Symptoms: Results from a Case-control Study

**DOI:** 10.7759/cureus.c20

**Published:** 2019-04-01

**Authors:** Ayman Fathy Abdelhaleim, Ahmed Y Amer, Jehan S Abdo Soliman

**Affiliations:** 1 Internal Medicine, Zagazig University Hospitals, Zagazig, EGY

The first author's name was erroneously entered as Jehan S Abdou Soliman (who is in fact the third and final author).

The correct first author has been added to the article: Ayman Fathy Abdelhaleim.

